# Curcumin-enhanced elvitegravir therapy mitigates neuroinflammation and cognitive deficits in EcoHIV mice

**DOI:** 10.3389/ebm.2025.10758

**Published:** 2025-11-07

**Authors:** Sandip Godse, Lina Zhou, Namita Sinha, Mohd Salman, Tauheed Ishrat, Santosh Kumar

**Affiliations:** 1 Department of Pharmaceutical Sciences, College of Pharmacy, University of Tennessee Health Science Center, Memphis, TN, United States; 2 Department of Anatomy and Neurobiology, College of Medicine, The University of Tennessee Health Science Center, Memphis, TN, United States

**Keywords:** HIV-associated neurocognitive disorders, EcoHIV, curcumin, elvitegravir, neuroinflammation

## Abstract

HIV-associated neurocognitive disorders (HAND) persist in up to 50% of people living with HIV (PLWH) despite effective antiretroviral therapy (ART), driven by chronic neuroinflammation, oxidative stress, and neuronal damage. This study investigates the therapeutic potential of combining elvitegravir (EVG), an integrase strand transfer inhibitor, with curcumin (CUR), a natural polyphenol with anti-inflammatory and antioxidant properties, in a murine EcoHIV model of HAND. EcoHIV-infected mice were treated with EVG, CUR, or their combination (EVG + CUR), and cognitive, motor, and molecular outcomes were evaluated. Behavioral assays revealed that EcoHIV infection significantly impaired non-spatial working memory, spatial learning, and motor performance, as assessed by the Novel Object Recognition (NOR)and Morris water Maize (MWM) tests and CatWalk gait analysis. While EVG or CUR alone showed modest improvements, the EVG + CUR combination significantly restored cognitive function, reduced escape latencies in the MWM, and improved motor performance, including gait stability and interlimb coordination. At the molecular level, EVG + CUR treatment attenuated neuroinflammation by reducing pro-inflammatory cytokines (IL-6, TNF-α, IL-1β) and chemokine (MCP-1) in the brain and plasma, particularly following intranasal administration. Additionally, EVG + CUR significantly reduced oxidative DNA damage and preserved neuronal integrity without disrupting CNS homeostasis. These findings demonstrate that the EVG + CUR combination effectively targets both viral persistence and the underlying neuroinflammatory and oxidative mechanisms driving HAND. By improving cognitive and motor function while mitigating neuroinflammation and oxidative stress, EVG + CUR represents a promising adjunctive therapy for HAND, offering a multifaceted approach to addressing the complex pathophysiology of HIV-associated neurocognitive disorders.

## Impact statement

Adjunctive strategies that safeguard neural integrity while suppressing HIV remain an unmet need because more than half of people living with HIV on combination antiretroviral therapy still develop neurocognitive impairment. We show that pairing the integrase inhibitor elvitegravir with the pleiotropic antioxidant curcumin restores cognitive performance, motor coordination, and neuronal marker expression in mice infected with ecotropic HIV-1, while concurrently dampening neuroinflammation, oxidative stress, and glutamate dysregulation—hallmarks of HIV-associated neurocognitive disorder (HAND). Molecular analyses confirmed preserved neuronal homeostasis and reduced pro-inflammatory signaling. These data provide the first preclinical evidence that curcumin can serve as an adjuvant to antiretroviral therapy to enhance neurotherapeutic efficacy. By simultaneously engaging antiviral and neuroimmune pathways with clinically relevant agents, this study charts a rapid translational path toward first-in-class combination interventions for HAND.

## Introduction

Human immunodeficiency virus (HIV) continues to pose a global public health burden despite the widespread use of combination antiretroviral therapy (cART) [1]. While cART has markedly reduced morbidity and mortality by suppressing systemic viral replication, up to 50% of people living with HIV (PLWH) still experience HIV-associated neurocognitive disorders (HAND), ranging from mild cognitive impairment to severe dementia [2, 3]. HAND significantly impairs quality of life and daily functioning [4], and its persistence despite virologic suppression highlights the need to understand and address its underlying mechanisms.

The pathophysiology of HAND is multifactorial and involves chronic neuroinflammation, oxidative stress, and synaptic and neuronal injury [5]. HIV enters the central nervous system (CNS) early in infection and establishes long-lived viral reservoirs in resident macrophages, microglia, and astrocytes [5]. Although cART reduces systemic viral load, many antiretroviral drugs have limited blood-brain barrier (BBB) permeability, allowing residual viral activity and inflammatory cascades to persist in the CNS [5, 6]. This persistent immune activation promotes the release of pro-inflammatory cytokines such as interleukin-6 (IL-6) and tumor necrosis factor-alpha (TNFα), and chemokine monocyte chemoattractant protein-1 (MCP-1), which contribute to neuronal dysfunction, synaptic loss, and behavioral deficits [5]. Additionally, oxidative stress, resulting from excessive production of reactive oxygen species (ROS), exacerbates neuronal injury by inducing mitochondrial damage and DNA oxidation [7].

Despite extensive research, current adjunctive treatments targeting neuroinflammation and oxidative stress in HAND remain limited. Several pharmacological interventions, including minocycline, memantine, and N-acetylcysteine, have demonstrated partial efficacy in preclinical models but have not translated into effective clinical strategies, in part due to poor CNS bioavailability or limited neuroprotective potency [8–11]. Thus, there is a critical need for therapeutic agents that can both enhance CNS delivery and mitigate neuroimmune and oxidative injury in HAND.

Curcumin (CUR), a natural polyphenol derived from turmeric (*Curcuma longa*), has emerged as a promising neuroprotective compound [12]. CUR possesses potent antioxidant and anti-inflammatory properties and has demonstrated efficacy in reducing ROS, inhibiting nuclear factor-kappa B (NF-κB) signaling, and enhancing synaptic plasticity in various models of neurodegeneration and inflammation [12, 13]. However, its clinical translation is hindered by poor oral bioavailability and limited BBB penetration [13, 14]. Intranasal (IN) delivery of therapeutic agents offers a non-invasive route that bypasses the BBB and enables direct nose-to-brain drug transport, thereby enhancing CNS drug targeting and efficacy [15].

In the present study, we investigated the neurotherapeutic potential of combining CUR with elvitegravir (EVG), a clinically approved integrase strand transfer inhibitor [16], in the EcoHIV murine model of HAND. This model recapitulates key pathological features of HAND, including CNS viral persistence, neuroinflammation, oxidative stress, and behavioral impairments [17–19]. We hypothesized that adjunctive administration of CUR with EVG would yield synergistic neuroprotective effects by simultaneously suppressing viral replication and attenuating inflammatory and oxidative injury.

Therefore, the present study was designed to evaluate whether combination therapy with EVG and CUR synergistically ameliorate EcoHIV-induced cognitive and motor impairments by attenuating neuroinflammation and oxidative stress. In addition, we compared IN and IP routes of administration to determine the impact of delivery strategy on therapeutic efficacy.

## Materials and methods

### Materials

Elvitegravir (EVG, E509000) was obtained from Toronto Research Chemicals, Inc. (North York, ON, Canada) and curcumin (CUR, 78246) from Sigma-Aldrich (St. Louis, MO, United States). Sterile phos-phate-buffered saline (PBS) (10100-031) was sourced from Gibco (Dublin, Ireland).

### Mice

Male and female C57BL/6J mice (6 weeks old) were procured from Jackson Laboratory (Bar Harbor, MA, United States) and acclimatized for 1 week prior to the study. Mice were housed under standard laboratory conditions (12-h light/dark cycle, controlled temperature and humidity) with *ad libitum* access to food and water. All experimental procedures were approved by the Institutional Animal Care and Use Committee (UTHSC-IACUC protocol #23-0464.0) and conformed to the NIH Guide for the Care and Use of Laboratory Animals. The study adhered to ARRIVE guidelines.

### EcoHIV infection, drug treatment, and plasma and brain harvesting

EcoHIV-NDK virus was generated as previously described [17, 20]. Briefly, 293T cells were transfected with the EcoHIV-NDK plasmid (obtained from Dr. David J. Volsky, Icahn School of Medicine at Mount Sinai) using a calcium phosphate transfection method. Viral supernatant was harvested 48 h post-transfection, filtered through a 0.45 µm membrane, and concentrated by ultracentrifugation at 25,000 rpm for 2 h at 4 °C. Viral titer was quantified by p24 antigen ELISA (Advanced Bioscience Laboratories, MD, United States). Virus stocks were aliquoted and stored at −80 °C until use.

**FIGURE 1:**
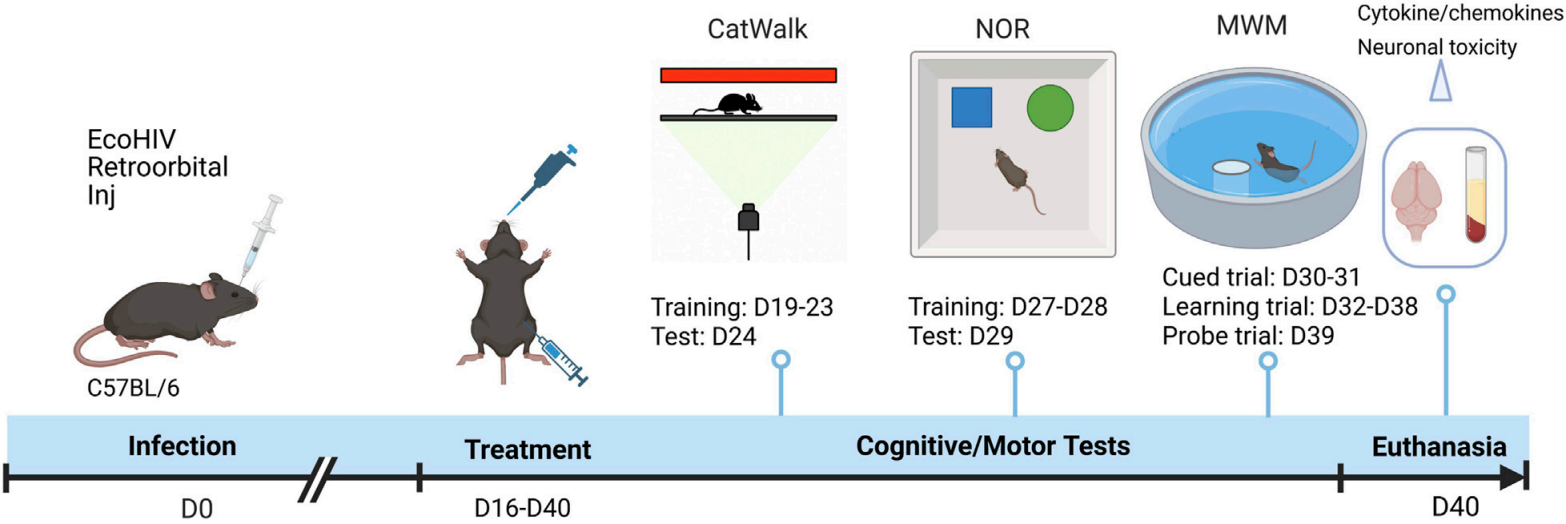
Experimental timeline illustrating EcoHIV infection, treatment, and behavioral assessment schedule. C57BL/6 mice were retro-orbitally inoculated with EcoHIV on Day 0 (D0). Treatment with EVG and CUR was initiated from Day 16 (D16) to Day 40 (D40). Motor function was evaluated using CatWalk gait analysis (training: D19–D23; test: D24). Cognitive function was assessed by NOR test (training: D27–D28; test: D29) and MWM test, comprising cued trials (D30–D31), learning trials (D32–D38), and a probe trial (D39). On D40, animals were euthanized, and brain and plasma samples were collected for cytokine analysis and neuronal toxicity assessment.

Mice were anesthetized with isoflurane and retro-orbitally injected with EcoHIV-NDK (10 µg of p24 antigen per mouse) diluted in sterile saline. Body weight (Supplementary Figure S1) and clinical signs were monitored post-infection. At day 16 post-infection, mice were randomized into five groups and administered EVG (25 mg/kg) and/or CUR (20 mg/kg) either via IN or intraperitoneal (IP) routes for 15 consecutive days (Figure 1). The vehicle group received equivalent volumes of saline. The dosing regimen was selected based on previous pharmacokinetic and safety studies.

Mice were euthanized on Day 40 by CO_2_ asphyxiation followed by transcardial perfusion with phosphate-buffered saline (PBS). Plasma and brain tissues were collected and stored at −80 °C for subsequent analyses.

### CatWalk test

Gait dynamics were assessed post-infection using the CatWalk XT automated gait analysis system (Noldus Information Technology, Wageningen, Netherlands). This video-based system enables objective quantification of spatiotemporal gait parameters in rodents. The setup consists of an enclosed glass walkway illuminated with a green light source from below and a red ceiling light source to enhance body contour contrast. A high-speed camera mounted underneath the walkway captures the paw prints and locomotor activity of freely moving animals [21]. Prior to testing, mice were acclimatized to the CatWalk apparatus over five consecutive days (Days 19–23) to minimize anxiety and ensure consistent voluntary crossing. Each daily training session lasted 30–45 min. On the day of testing (Day 24), mice were allowed to traverse the walkway voluntarily without external stimulus. For each animal, a minimum of three compliant runs—defined as uninterrupted, straight, and consistent movement across the walkway—were recorded. The average of these runs was used for final analysis. The CatWalk XT software automatically analyzed the captured data to compute various gait parameters reflective of motor coordination and balance. The key gait parameters assessed included run maximum variation, which quantifies the variability in locomotor patterns across multiple runs; run average speed, representing the mean velocity of the animal during walkway traversal; and paw print area, indicating the contact surface area of each paw on the glass platform. Additionally, stand duration was measured to determine the time each paw remained in contact with the walkway, while the stand index reflected the distribution of weight borne by each paw during the stance phase. Support patterns were also analyzed to evaluate inter-paw coordination and limb support dynamics, providing insights into postural control and motor coordination. These parameters provided a comprehensive assessment of EcoHIV-induced motor deficits and therapeutic effects of treatment. Definitions of gait parameters analyzed using CatWalk are detailed in Supplementary Table S1.

### Novel object recognition test (NOR)

The NOR test was used to assess short-term recognition memory based on rodents’ natural preference for novel objects. The protocol was adapted from following the published method with minor modifications [20, 22]. On day 20 post-infection, animals were habituated to an open field arena (45 × 45 × 35 cm) for 10 min without objects. On the following day, two identical objects were placed in the arena during the acquisition trial, and animals were allowed to explore for 5 min. After a 90-min retention interval, one familiar object was replaced with a novel object, and the animals were reintroduced for a 5-min test trial. The time spent exploring each object was recorded, and the recognition index (RI) and discrimination index (DI) was calculated as:

$$\text{Recognition index }\left(\text{DI}\right)=\frac{\text{Novel}\;\text{Object}\;\text{Exploration}\;\text{Time}}{\text{Total}\;\text{Exploration}\;\text{Time}}$$

$$\text{Discrimination index }\left(\text{DI}\right)=\frac{\text{Novel}\;\text{Object}\;\text{Exploration}\;‐\;\text{Familiar}\;\text{Object}\;\text{Exploration}\;\text{Time}}{\text{Total}\;\text{Exploration}\;\text{Time}}$$

### Morris water maze (MWM) test

Spatial learning and memory were assessed using the MWM test, performed between Days 30–39 post-infection, following the published protocol [22]. The apparatus consisted of a circular pool (132 cm diameter, 60 cm height) filled with opaque water (25 ± 2 °C). A hidden escape platform (10 cm diameter) was submerged 2 cm below the water surface in one quadrant. Visual cues were placed around the testing room to aid spatial orientation.

The training phase comprised four trials per day with cued trial (Days 30-31) and learning trial over seven consecutive days (Days 32–38). In each trial, mice were released from one of four random starting points and allowed 60 s to locate the platform. If unsuccessful, mice were guided to the platform and allowed to remain for 30 s. Escape latency (time to locate the platform) was recorded. On Day 39, a probe trial was conducted to assess memory retention. The platform was removed, and mice were allowed to swim freely for 60 s. The percentage of time spent, and distance traveled in the target quadrant were recorded as indices of memory consolidation.

### Cytokine analysis

#### Brain lysate preparation

Following euthanasia and transcardial perfusion with ice-cold PBS, whole brains were rapidly removed, blotted, and weighed on ice. Tissue was homogenized at a 1:10 (w/v) ratio in ice-cold PBS containing 0.1% Triton X-100 and a protease inhibitor cocktail, keeping samples on ice throughout. Homogenates were clarified by centrifugation at 12,000 × g for 15 min at 4 °C, and the supernatant was collected as the brain lysate. Total protein concentration was determined by BCA assay (Thermo Scientific) and lysates were normalized to an equal protein concentration prior to cytokine quantification. Where needed, samples were further diluted per the manufacturer’s recommendations to fall within the dynamic range of the assay.

#### Plasma preparation

Blood was collected into EDTA-coated tubes at necropsy, kept on ice, and centrifuged at 1,500 × g for 10 min at 4 °C. The plasma supernatant was aliquoted and stored at −80 °C until analysis; samples underwent a single freeze–thaw cycle.

#### Multiplex cytokine quantification

Cytokine and chemokine levels, including proinflammatory markers (IL-1β, TNF-α, IL-8, IL-6, IL-18), anti-inflammatory markers (IL-1RA, IL-10), and chemokines (MCP-1, RANTES), were quantified in brain and plasma samples. A Mouse Custom Procartaplex 6-plex panel (Invitrogen, Thermo Fisher Scientific, Grand Island, NY, United States) was used for analysis according to the manufacturer’s protocol, as described in previous reports [23, 24]. Briefly, samples, standards, and fluorescent magnetic beads were loaded into 96-well plates, mixed on a shaker at room temperature for 1 h, and subsequently incubated overnight at 4 °C. Following incubation, extensive washing was performed, after which detection antibody, streptavidin–PE, and reading buffer were sequentially applied, with intermediate washing steps. Cytokine and chemokine concentrations (pg/mL) were acquired using the Magpix system and analyzed via xPONENT® 4.2 software.

### Quantification of oxidative DNA damage in brain tissue

Genomic DNA was extracted from 100 μL of mouse brain homogenate using the QIAamp DNA Mini Kit (QIAGEN, Germantown, MD, United States) according to protocol as described in our previous report [20]. DNA purity and concentration were assessed using a NanoDrop™ 2000 spectrophotometer (Thermo Scientific, Waltham, MA, United States) by measuring absorbance at 260/280 nm.

Oxidative DNA damage was evaluated by quantifying 8-hydroxy-2′-deoxyguanosine (8-OHdG), a well-established biomarker of oxidative lesions in DNA. Quantification was performed using the EpiQuik™ 8-OHdG DNA Damage Quantification Direct Kit (Colorimetric) (EPIGENTEK, Farmingdale, NY, United States), following the manufacturer’s protocol. In brief, isolated DNA was immobilized onto assay wells using a DNA binding solution and incubated with a capture antibody specific to 8-OHdG, followed by a detector antibody. Color development was achieved with a chromogenic substrate, and absorbance was measured at 450 nm using a microplate reader. The levels of 8-OHdG were expressed relative to total DNA content.

### Quantification of glutamate levels in mouse brain

Glutamate levels in brain homogenates were quantified using the Amplex® Red Glutamic Acid/Glutamate Oxidase Assay Kit (Invitrogen, A12221), following the manufacturer’s protocol. Briefly, brain tissue was homogenized in PBS, centrifuged at 12,000 × g for 10 min at 4 °C, and the supernatant was used for analysis. Samples and standards were incubated with a reaction mix containing Amplex® Red reagent, glutamate oxidase, HRP, and associated enzymes at 37 °C for 30 min. Fluorescence was measured at Ex/Em 530/590 nm, and glutamate concentrations were calculated using a standard curve.

### Western blotting

Western blotting was performed to evaluate the expression of neural markers in mouse brain tissue. Proteins (15 μg) were extracted from the brain homogenates of Non-EcoHIV controls, EcoHIV-infected mice, and treatment groups receiving EVG, CUR, or EVG + CUR. Protein samples were separated using SDS-PAGE with a 4% stacking gel and 10% resolving gel at 150 V for 90 min. After electrophoresis, the proteins were transferred onto a polyvinylidene fluoride (PVDF) membrane at a constant current of 0.35 A for 90 min. The membranes were subsequently blocked with Li-Cor blocking buffer (Li-Cor Biosciences, Lincoln, NE, United States) for 1 h at room temperature to reduce nonspecific binding. Primary antibody incubation was carried out overnight at 4 °C using the following antibodies: NeuN rabbit polyclonal antibody (1:1000, Proteintech, Cat# 26975-1-AP), synaptophysin mouse monoclonal antibody (1:20,000, Proteintech, Cat# 67864-1-Ig), GFAP rabbit polyclonal antibody (1:1000), L1CAM rabbit polyclonal antibody (Proteintech, Cat# 20659-1-AP), and β-actin mouse monoclonal antibody (1:20,000, Proteintech, Cat# 66009-1-Ig) as an internal loading control. The membranes were washed three times with PBST (PBS supplemented with 0.2% Tween-20) and then incubated for 1 h at room temperature with IRDye-labeled secondary antibodies: goat anti-mouse and goat anti-rabbit IgG (1:10,000, Li-Cor Biosciences). After final washes, the blots were visualized using a Li-Cor Odyssey imaging system, and band intensities were quantified using Image Studio Lite version 4.0 software. Densitometric values were normalized against β-actin levels to account for loading variability.

## Results

### Evaluation of gait alterations and motor performance following EVG and CUR treatment in EcoHIV-infected mice

**FIGURE 2:**
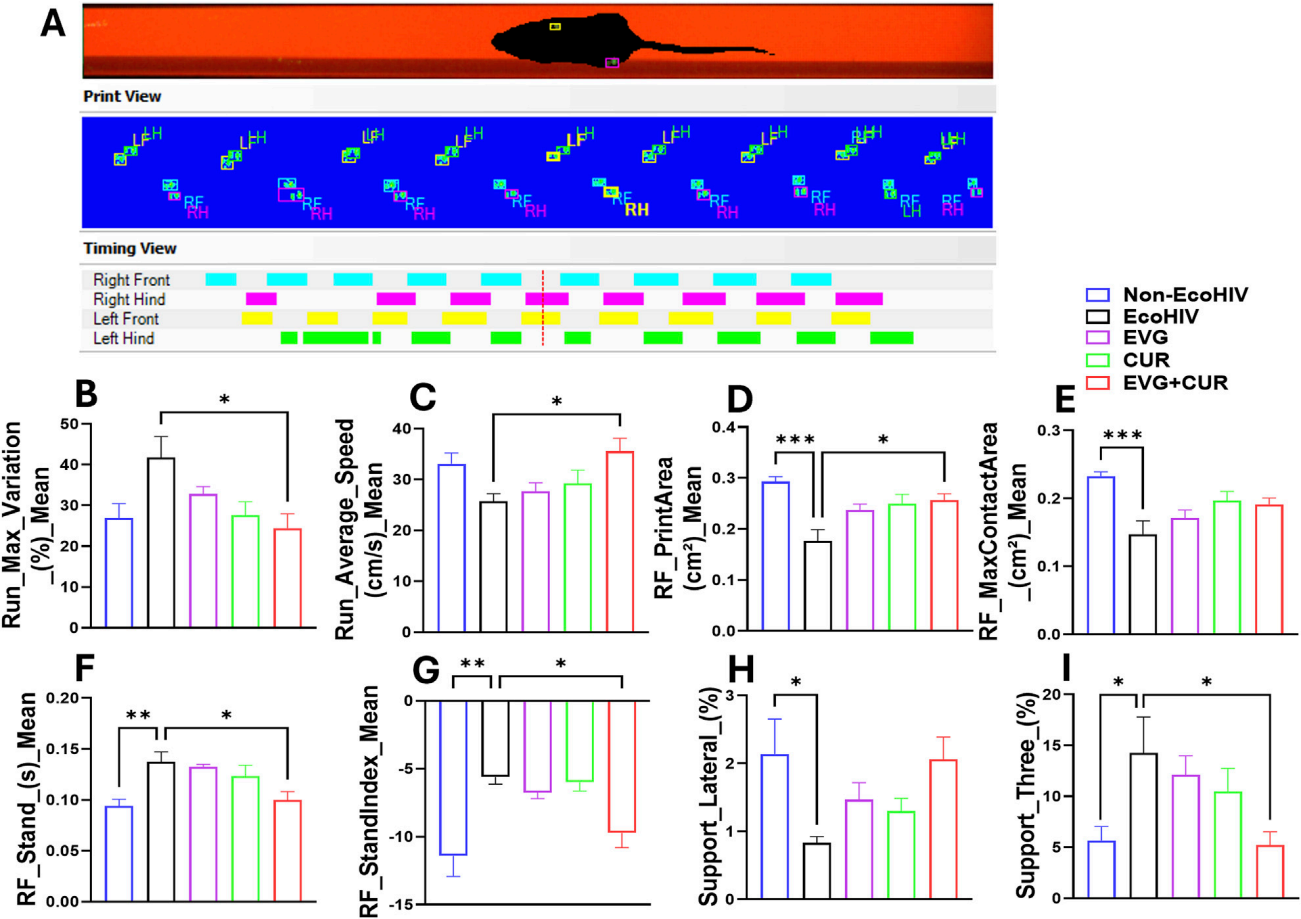
**(A–I)** Evaluation of gait using CatWalk test. **(A)** Represent experimental set and the basic measures of animal’s footprints on the walkway. **(B–I)** Gait control indices based on the footprints. One-way ANOVA with Tukey’s *post hoc* test was applied to compare between multiple groups. Data are presented as mean ± SEM (*n* = 8). *, **, and *** represent p < 0.05, p ≤ 0.01, and p ≤ 0.001 respectively.

To evaluate the impact of EcoHIV infection and the therapeutic effect of EVG, CUR, and their combination on gait control, several parameters were analyzed using CatWalk gait assessment. EcoHIV-infected mice exhibited significant impairments in multiple gait indices compared to uninfected controls (Figures 2C-I). Run max variation was notably increased in the EcoHIV group, indicating stride inconsistency, while treatment with EVG and CUR, particularly in combination, significantly lowered this variation, restoring gait stability (Figure 2C, *p < 0.05). RF stand time was increased in EcoHIV-infected mice (Figure 2G, **p < 0.01), suggesting altered weight-bearing balance, whereas EVG + CUR combination treatment reduced stand time compared to the EcoHIV control (Figure 2G, *p < 0.05). Similarly, RF print area, a measure of paw contact stability, was significantly lower in EcoHIV mice (Figure 2E, *p < 0.05) but improved with EVG + CUR treatment (Figure 2E, *p < 0.05), with both individual treatments showing a similar trend, albeit without statistical significance. In addition, RF stand index was elevated in EcoHIV mice (Figure 2H, **p < 0.01), indicating an imbalance in weight distribution, but was effectively reduced by EVG + CUR treatment (Figure 2H, *p < 0.05). However, EVG or CUR alone did not significantly affect this parameter. Lateral support phase, which measures interlimb coordination, was significantly decreased in EcoHIV mice (Figure 2I, *p < 0.05) and showed an increasing trend with EVG + CUR, though it did not reach statistical significance. Finally, run average speed, a measure of locomotor efficiency, was lower in EcoHIV-infected mice but significantly improved following EVG + CUR treatment (Figure 2D, *p < 0.05), indicating enhanced overall motor performance.

### Evaluation of EVG + CUR effects on EcoHIV-associated cognitive impairment using NOR and MWM

**FIGURE 3:**
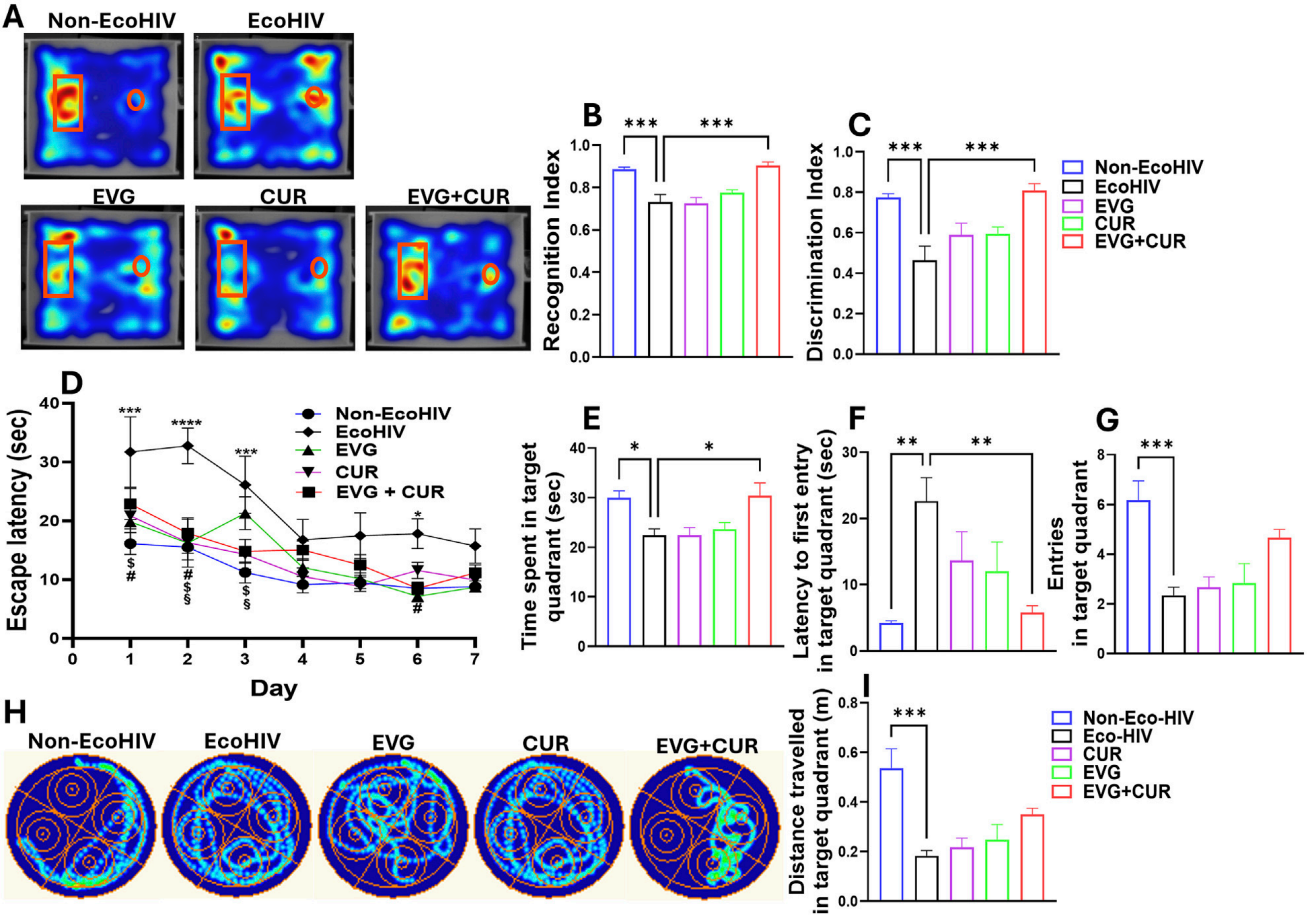
Evaluation of cognitive performance in EcoHIV mice. **(A–C)** Assessment of short-term recognition memory using the NOR test. **(A)** Representative heatmaps illustrate exploratory preference toward the novel (rectangle) and familiar (circle) objects across treatment groups. **(B)** Recognition index (RI) and **(C)** Discrimination index (DI) evaluating non-spatial working memory in different treatment groups. **(D–I)** Spatial learning and memory were evaluated using the MWM test. **(D)** Escape latency time in the target quadrant during the consecutive 7 days. **(H)** Representative heatmap showing the swim path across experimental groups. Different cognitive indices represented as **(B)** time spent in target quadrant, **(D)** target quadrant entry, **(F)** distance travelled in target quadrant and increased **(C)** latency to first entry in target quadrant. One-way ANOVA with Tukey’s *post hoc* test was applied to compare between multiple groups. Data are presented as mean ± SEM (*n* = 8). *, **, ***and **** represent p < 0.05, p ≤ 0.01, p ≤ 0.001, and p ≤ 0.0001 respectively. Two-way ANOVA with Tukey’s *post hoc* test was applied to compare between multiple groups (Figure 2D). ^#^p < 0.05 (EVG Vs. EcoHIV), ^$^p < 0.05 (CUR Vs. EcoHIV), ^§^p < 0.05 (EVG + CUR Vs. EcoHIV).

To investigate EcoHIV-induced deficits in short-term recognition memory and assess therapeutic interventions, mice underwent the NOR (Novel object recognition test) test (Figures 3A–C). Heatmap analyses revealed exploration patterns, with greater engagement toward the novel object observed in Non-EcoHIV and EVG + CUR-treated mice (Figure 3A). Recognition index (RI) and Discrimination index (DI) were determined for each mouse, and the mean values were compared across groups. Quantitative analysis revealed significant reductions in the RI (Figure 3B, ***p < 0.001) and DI (Figure 3C, ***p < 0.001) in EcoHIV-infected mice compared to uninfected controls, confirming impaired non-spatial working memory. Treatment with either EVG or CUR alone did not significantly ameliorate these deficits. However, combination therapy with EVG + CUR significantly improved both RI and DI indices, restoring performance towards Non-EcoHIV controls (Figures 3A,B, ***p < 0.001). These results suggest that the combined antiviral and anti-inflammatory actions of EVG and CUR effectively mitigate EcoHIV-associated impairments in short-term recognition memory.

The Morris Water Maze (MWM) test was conducted to evaluate the impact of EcoHIV infection and treatment with EVG, CUR, or their combination (EVG + CUR) on spatial learning, long-term/reference memory, and cognitive flexibility (Figures 3D–I). After 30 days of post-infection, escape latency was calculated for 7 days of the hidden platform. Four trials per day were recorded and considered to determine spatial learning memory. EcoHIV-infected mice exhibited significantly prolonged escape latencies compared to non-EcoHIV controls specifically on day 1, 2, 3 and day 6 (Figure 3D, *p < 0.05, ***p < 0.001, ****p < 0.0001), indicating deficits in spatial learning. While treatment with EVG showed improvements in spatial learning at day 1, 2 and 6 (Figure 3D ^#^p < 0.05), CUR alone treatment showed improvements at day 1, 2, and 3 (Figure 3D ^$^p < 0.05). EVG + CUR combination significantly reduced escape latency specifically at day 2 and 3 (Figure 3D ^§^p < 0.05), suggesting enhanced learning ability across the treatment groups. Other cognitive indices derived from the probe trial such as time spent (Figure 3E), latency to first entry (Figure 3F), number of entries (Figure 3G) and distance travelled (Figure 3I) in target quadrant show significant deficit in EcoHIV group when compared to Non-EcoHIV control indicating impaired long-term memory retention. The combination treatment of EVG + CUR partially restored memory performance, as reflected by increased time spent in the target quadrant (Figure 3E, *p < 0.05) and reduced latency to first entry (Figure 3F, **p < 0.01). EcoHIV mice exhibited significantly fewer entries into the target quadrant and reduced distance traveled, suggesting impaired cognitive flexibility and spatial navigation, which were partially restored by EVG + CUR treatment though it didn’t reach the statistical significance (Figures 3G,I). Overall, these findings suggest that the combination of EVG and CUR enhances cognitive function, spatial learning, and motor performance, mitigating EcoHIV-induced impairments more effectively than either treatment alone.

### Profiling of EVG + CUR effects on neuroinflammatory cytokine levels in brain lysates of EcoHIV-infected mice

**FIGURE 4:**
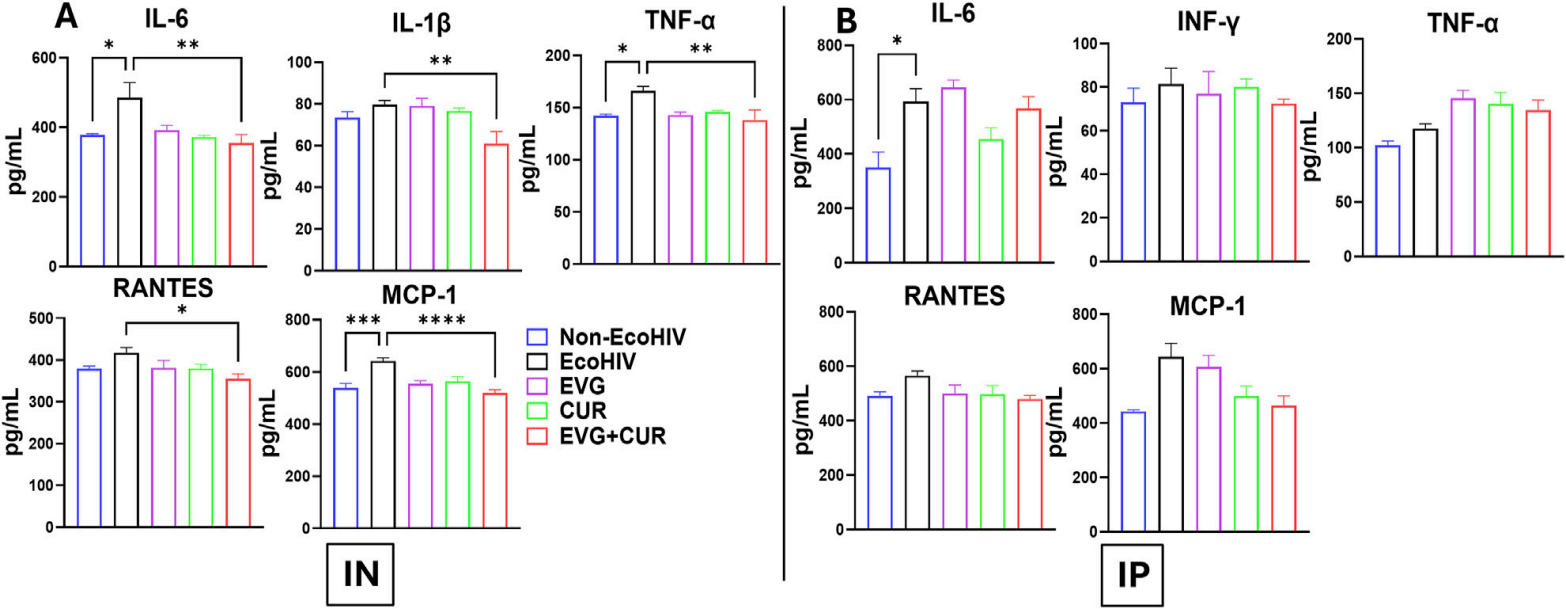
Effect of EVG and CUR combinationon cytokine levels in C57BL/6 mouse brain **(A)** IN route and **(B)** IP route of treatment. Brain cytokine and chemokine concentrations, including IL-6, IFN-γ, TNF-α, RANTES, and MCP-1, were measured using a Mouse Custom Procartaplex multiplex ELISA kit (Invitrogen, Thermo Fisher Scientific). Statistical analysis among multiple groups was conducted using one-way ANOVA followed by Tukey’s *post hoc* test. Data are presented as mean ± SEM (n = 6 per group). *, **, ***, and **** represents p ≤ 0.05, p ≤ 0.01, p ≤ 0.001, and p ≤ 0.0001, respectively.

The effect of EVG in the presence of CUR on cytokine modulation in the brain of EcoHIV C57BL/6 mice was assessed following IN and IP administration. EcoHIV infection significantly elevated pro-inflammatory cytokines, including IL-6, IL-1β, TNF-α, and chemokines RANTES and MCP-1, indicating heightened neuroinflammation. IN administration (Figure 4A) of EVG + CUR significantly reduced IL-6 (**p ≤ 0.01), TNF-α (**p ≤ 0.01), IL-1β (**p ≤ 0.01), and MCP-1 (****p ≤ 0.0001), with a moderate decrease in RANTES (*p ≤ 0.05) compared to EcoHIV control. In contrast, IP administration (Figure 4B) of EVG + CUR did not result in significant reductions in inflammatory cytokines, suggesting a lesser impact of systemic administration on brain inflammation. These findings highlight the superior efficacy of IN EVG + CUR administration in attenuating neuroinflammation in EcoHIV-infected mice, reinforcing its potential for mitigating HIV-associated neuropathogenesis.

### Profiling of effects of EVG + CUR on systemic cytokine levels in plasma samples of EcoHIV-infected mice

**FIGURE 5:**
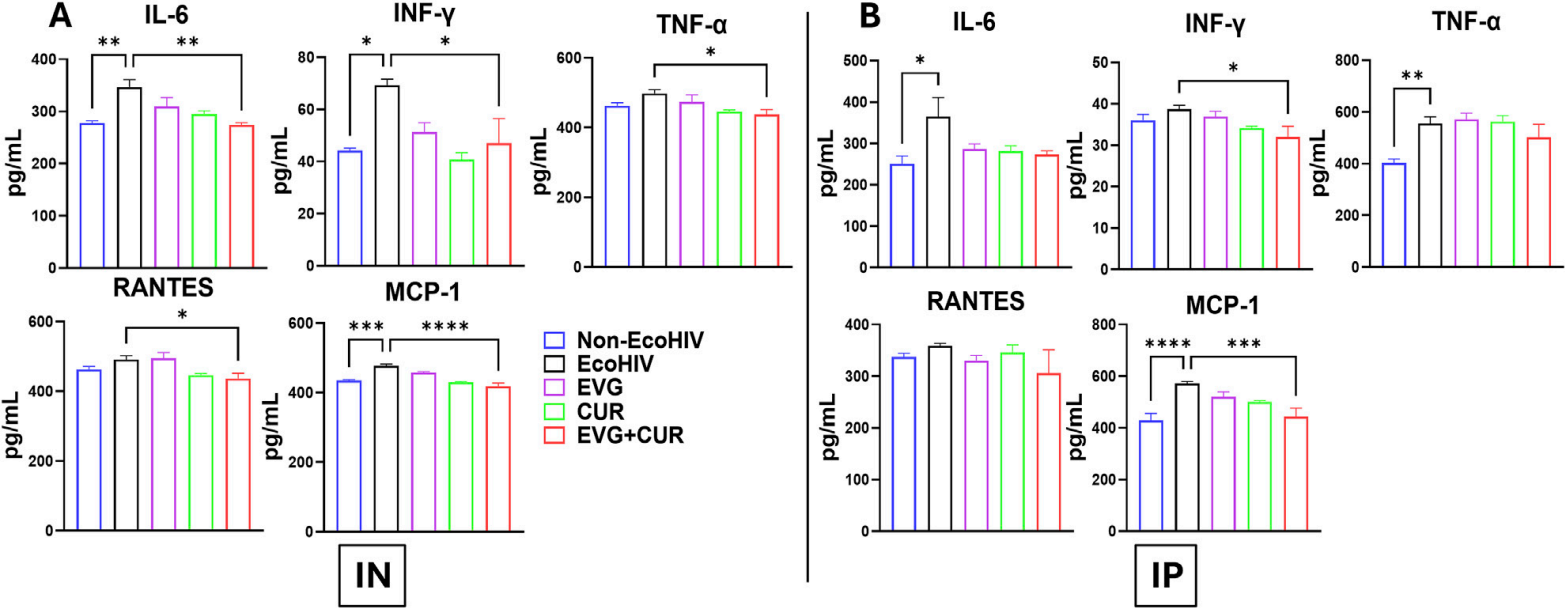
Effect of EVG and CUR combination on cytokine levels in C57BL/6 mouse plasma. Plasma cytokine and chemokine concentrations, including IL-6, IFN-γ, TNF-α, RANTES, and MCP-1, were measured using a Mouse Custom Procartaplex multiplex ELISA kit (Invitrogen, Thermo Fisher Scientific). Statistical analysis among multiple groups was conducted using one-way ANOVA followed by Tukey’s *post hoc* test. Data are presented as mean ± SEM (n = 6 per group). *, **, ***, and **** represents p ≤ 0.05, p ≤ 0.01, p ≤ 0.001, and p ≤ 0.0001, respectively.

The effect of EVG in the presence of CUR on cytokine modulation in EcoHIV C57BL/6 mouse plasma was assessed following IN and IP administration. The results indicate that EcoHIV infection led to increased levels of pro-inflammatory cytokines IL-6, INF-γ, TNF-α, and chemokines RANTES and MCP-1 in plasma. IN administration (Figure 5A) of EVG + CUR significantly reduced IL-6 (**p ≤ 0.01), INF-γ (*p ≤ 0.05), TNF-α (*p ≤ 0.05), RANTES (*p ≤ 0.05) and MCP-1 (****p ≤ 0.0001) levels compared to EcoHIV control. In contrast, IP administration (Figure 5B) of EVG + CUR exhibited a moderate reduction in cytokines, with significant decreases in INF-γ (*p ≤ 0.05) and MCP-1 (***p ≤ 0.001). These findings suggest that the combination treatment of EVG + CUR, particularly via IN administration, effectively attenuates systemic inflammation in EcoHIV-infected mice.

### Determination of oxidative stress, glutamate levels, and CNS homeostasis following EVG + CUR treatment in EcoHIV-infected mice

**FIGURE 6:**
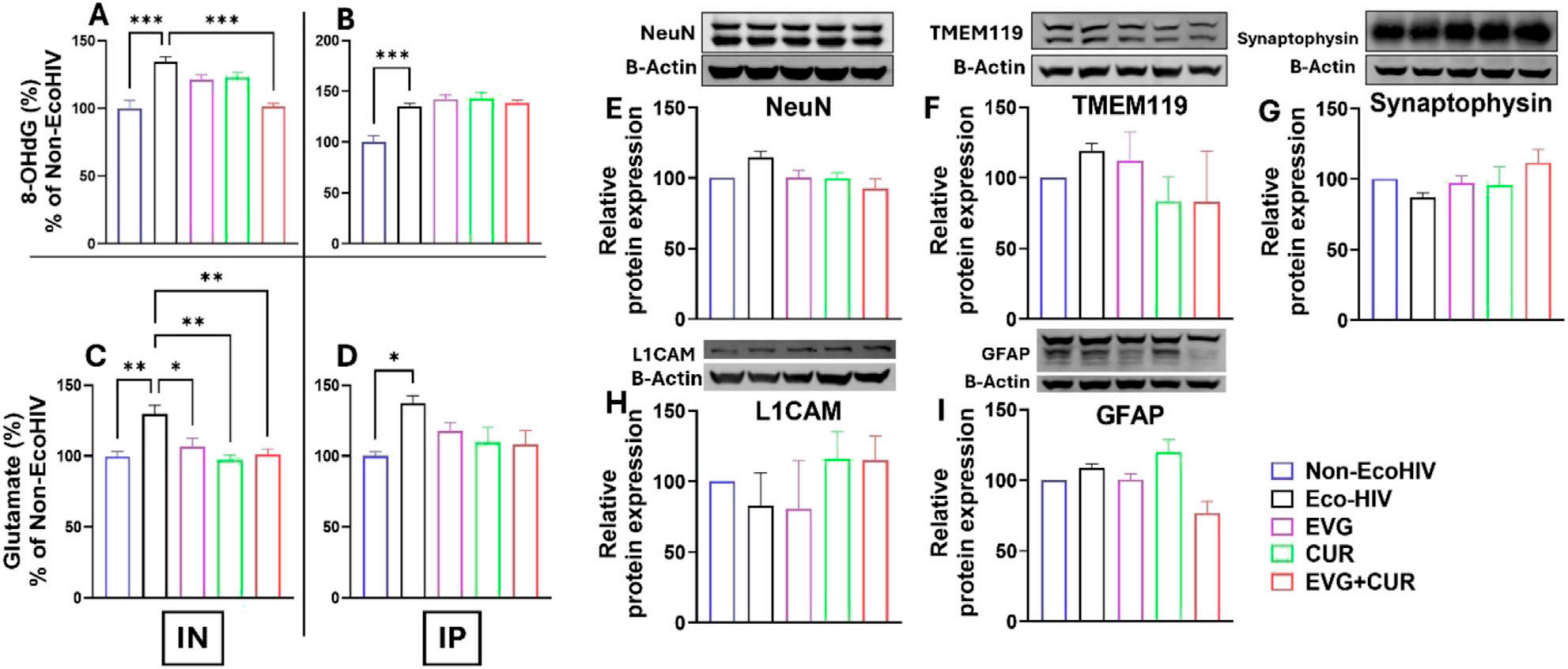
Brain levels of 8-OHdG **(A,B)** and glutamate **(C,D)** were measured in EcoHIV-infected mice treated with EVG, CUR, or their combination (EVG + CUR) via IN or IP routes. **(E–I)** Western blot analysis was conducted to assess the expression of neural protein markers NeuN, TMEM119, synaptophysin, L1CAM, and GFAP in the mice brains. Data are expressed as mean ± SEM (n = 4 per group). Statistical analysis was performed by one-way ANOVA with Tukey’s *post hoc* test. *, **, and *** represents p ≤ 0.05, p ≤ 0.01, and p ≤ 0.001 respectively.

Oxidative DNA damage was assessed by quantifying 8-hydroxy-2′-deoxyguanosine (8-OHdG) levels in the brains of EcoHIV-infected mice (Figures 6A,B). EcoHIV infection significantly increased 8-OHdG levels compared to Non-EcoHIV controls (***p ≤ 0.001), indicative of heightened oxidative stress. Following IN administration treatment with EVG or CUR alone produced only modest reductions in 8-OHdG levels. However, combination therapy with EVG + CUR significantly decreased oxidative DNA damage relative to the EcoHIV group (Figure 6A ***p ≤ 0.001). On the other hand, IP delivery did not result in reductions in any treatment group (Figure 6B). Glutamate concentrations were also measured to assess excitotoxic stress (Figures 6C,D). EcoHIV infection led to a significant elevation in brain glutamate levels in both IN (Figure 6C **p ≤ 0.01) and IP (Figure 6D *p ≤ 0.05) cohorts compared to Non-EcoHIV controls. In the IN-treated groups, EVG, CUR, and EVG + CUR therapies substantially reduced glutamate concentrations (Figure 6C *p ≤ 0.05, **p ≤ 0.01), approaching levels observed in uninfected mice. Notably, the CUR-only and EVG + CUR groups exhibited the most pronounced reductions. In contrast, IP administration (Figure 6D) did not significantly lower elevated glutamate levels in any treatment group.

The effect of EVG in the presence of CUR on the modulation of homeostatic CNS markers was assessed in EcoHIV-infected C57BL/6 mice. The expression levels of key neural markers, including NeuN (Figure 6E), TMEM119 (Figure 6F), Synaptophysin (Figure 6G), L1CAM (Figure 6H), and GFAP (Figure 6I), were evaluated to determine the impact of treatment on neural homeostasis. Western blot analysis revealed that EcoHIV infection did not significantly alter the expression of these markers compared to the non-EcoHIV control group. Similarly, treatment with EVG, CUR, or their combination did not induce significant changes in the expression levels of NeuN (a neuronal marker), TMEM119 (a microglial marker), Synaptophysin (a synaptic marker), L1CAM (a neural adhesion molecule), or GFAP (an astrocytic marker). These findings suggest that EVG and CUR, either alone or in combination, do not disrupt CNS homeostasis, further supporting the safety of the EVG + CUR treatment in preserving neuronal integrity and function in the context of EcoHIV infection.

## Discussion

This study demonstrates that the combination of EVG and CUR significantly ameliorates cognitive and motor impairments, reduces neuroinflammatory cytokine expression, and attenuates oxidative DNA damage in an EcoHIV-infected murine model of HAND. These findings provide the first preclinical evidence supporting the utility of CUR as an adjunct to antiretroviral therapy (ART) in addressing the persistent neuroimmune and oxidative pathologies that underlie HAND, particularly when delivered via the IN route to enhance CNS targeting.

Behavioral assessments revealed that EcoHIV infection impaired recognition memory, spatial learning, and motor coordination, as measured by NOR, MWM, and CatWalk gait analysis, respectively. These deficits align with previously reported cognitive and motor impairments in EcoHIV and gp120-transgenic rodent models [18–20, 25, 26]. ART has been reported to partially improve cognitive performance in rodent models; however, cognitive flexibility, reference memory, and recognition memory deficits often persist [27, 28]. Similarly, adjunctive interventions using neuroprotective or anti-inflammatory agents, such as minocycline and memantine, have yielded modest cognitive improvements [9, 29]. In our study, EVG or CUR monotherapy conferred limited behavioral benefits. In contrast, the combination therapy resulted in significant improvements in both MWM and NOR performance.

In addition to cognitive assessments, this study employed CatWalk XT gait analysis to quantify motor deficits in a HAND model. To our knowledge, our group is among the first to employ CatWalk XT gait analysis to quantitatively assess motor deficits in a murine model of HAND, as demonstrated in our earlier publications [20, 21]. Building on this prior work, the current study leveraged CatWalk to enable objective evaluation of gait parameters, providing novel insights into HIV-associated motor dysfunction and the therapeutic efficacy of EVG + CUR treatment. Previous preclinical studies have primarily focused on cognitive endpoints, with limited evaluation of motor impairments despite their clinical relevance in PLWH [18, 19, 30]. Our use of CatWalk provides objective, sensitive, and quantitative measures of gait abnormalities, establishing a novel behavioral outcome for assessing HAND-associated motor dysfunction and therapeutic response. The results demonstrated that EcoHIV infection induced significant abnormalities in gait parameters, including increased run maximum variation and reduced right forelimb stand time, indicative of impaired coordination and postural instability. Treatment with EVG + CUR normalized these parameters, highlighting the therapeutic efficacy of the combination in mitigating HIV-associated motor dysfunction.

A prominent feature of HAND is chronic neuroinflammation driven by activated microglia and astrocytes [31, 32]. Results showed that EcoHIV infection elevated brain levels of IL-6, TNF-α, IL-1β, and MCP-1, consistent with clinical reports of persistent cytokine dysregulation in ART-treated individuals [33–35]. EVG + CUR therapy markedly reduced these inflammatory mediators, with IN administration achieving greater reductions than IP delivery, likely reflecting improved CNS bioavailability. CUR is known to exert its anti-inflammatory and antioxidant effects via modulation of NF-κB, Nrf2/ARE signaling, and inhibition of MAPK pathways, as well as by enhancing mitochondrial bioenergetics and limiting ROS production [12, 36, 37]. These mechanistic insights support the observed reductions in pro-inflammatory cytokines and oxidative stress by CUR and the combination therapy. Although EVG is expected to reduce viral burden, we did not directly quantify EcoHIV RNA/DNA in brain or plasma by RT-qPCR in this study. Therefore, the behavioral and molecular improvements observed with EVG + CUR cannot be attributed solely to changes in viral load. This design choice reflects our focus on neuroimmune and oxidative endpoints that are central to HAND pathophysiology and are known to persist even when systemic viremia is suppressed. The persistence of HAND despite virologic control has been widely reported, including evidence for residual brain reservoirs during ART, underscoring the importance of targeting neuroinflammation and oxidative injury in parallel with antiretroviral therapy.

In addition to CNS inflammation, systemic immune activation is increasingly recognized as a contributor to HAND [32, 38]. Elevated plasma cytokines, including IL-6, TNF-α, and MCP-1, have been implicated in BBB disruption and peripheral immune cell trafficking into the brain [39–41]. The study showed that EVG + CUR also reduced circulating cytokine levels, indicating peripheral anti-inflammatory effects that may further support CNS protection. Our findings align with clinical studies reporting elevated plasma cytokines in ART-treated PLWH, which correlate with cognitive impairment and comorbidities [42, 43]. Previous preclinical studies have similarly demonstrated that cART reduces, but does not normalize, systemic inflammation in HIV-infected rodents [44, 45]. The significant reduction in plasma cytokines observed with EVG + CUR treatment thus suggests a broader systemic immune modulatory effect beyond CNS protection. This dual central and peripheral immune modulation highlights the broader therapeutic potential of combination therapy.

Glutamate excitotoxicity is a key driver of neuronal injury in HAND, linked to oxidative stress and synaptic dysfunction [5, 46]. In this study, EcoHIV-infected mice showed elevated brain glutamate levels, which were significantly reduced by CUR, especially via IN administration. Notably, CUR alone and in combination with EVG produced the greatest reductions, suggesting CUR’s potential role in modulating glutamate clearance, possibly through astrocytic or transporter-mediated mechanisms [47]. These reductions corresponded with improvements in cognitive performance, decreased oxidative DNA damage, and suppressed neuroinflammation, underscoring the central role of glutamate dysregulation in HAND pathogenesis and the therapeutic relevance of CUR-based interventions.

Oxidative stress has been implicated as a key mechanism in HIV-associated neuronal injury [48]. Elevated brain levels of 8-OHdG, a biomarker of oxidative DNA damage, were observed in EcoHIV-infected mice, consistent with reports from both clinical and preclinical studies [7, 49, 50]. While EVG or CUR alone had minimal impact on 8-OHdG levels, the combination therapy significantly reduced oxidative damage, suggesting that CUR’s antioxidant properties synergize with EVG’s antiviral effects to restore redox balance. IN delivery of EVG + CUR potentiated this effect, emphasizing the importance of optimized drug delivery in neuroHIV therapy.

Notably, EVG + CUR treatment did not alter the expression of markers for neurons (NeuN), microglia (TMEM119), astrocytes (GFAP), or synapses (Synaptophysin), suggesting that the therapy does not adversely affect CNS cellular composition or homeostasis. This observation is consistent with prior reports showing that neuronal marker alterations in chronic HAND models often reflect advanced neurodegeneration [51, 52]. The preserved expression of NeuN, TMEM119, Synaptophysin, and GFAP in our short-term study underscores the early-stage nature of HAND pathology captured here and supports the safety of EVG + CUR therapy. This supports the safety of the combination approach and indicates that behavioral improvements likely reflect functional restoration rather than compensatory structural changes.

The observed synergistic effect of EVG + CUR is likely multifactorial. While EVG primarily reduces viral burden, CUR targets inflammatory and oxidative pathways. It is plausible that this dual action also affects the neurovascular unit by preserving tight junction proteins and mitigating endothelial activation, thereby maintaining BBB integrity. Additionally, CUR has been reported to upregulate neurotrophic factors such as BDNF and modulate synaptic plasticity, which could contribute to behavioral improvements [53]. Furthermore, since CUR is known to inhibit BBB efflux transporters [54, 55], it is likely that CUR increases EVG permeability in the CNS ehhancing its antiviral effect. In fact, we have recently shown that addition of CUR to EVG increases its brain distribution relative to control via both IP and IN routes [23]. Future studies incorporating transcriptomic or proteomic profiling may further elucidate these synergistic mechanisms and help identify specific molecular interactions.

Importantly, IN delivery conferred superior therapeutic outcomes compared to IP administration, likely due to enhanced CNS bioavailability via the olfactory and trigeminal nerve pathways [56]. Optimizing delivery systems, such as using mucoadhesive or nanoparticle-based formulations, could further improve brain targeting [57]. IN delivery is gaining traction in clinical research, and several early-phase trials are underway evaluating this route for neuroactive compounds in neuroinflammatory disorders [58, 59], highlighting its translational potential. Additional supporting evidence from the literature further contextualizes our results. The EcoHIV-induced cognitive and motor phenotypes we observe mirror prior reports in EcoHIV and gp120 models [4, 18, 19, 25, 26, 30], and our use of CatWalk® to quantify gait abnormalities builds on earlier methodological work from our group and others [20, 21]. The superiority of intranasal administration that we demonstrate is consistent with established nose-to-brain delivery principles and emerging translational studies evaluating this route [15, 56–59].

Mechanistically, the combination of EVG and CUR is supported by published data showing CUR’s anti-inflammatory and antioxidant actions [12, 36, 37] and by our prior work demonstrating that CUR enhances EVG brain distribution and modulates inflammatory/oxidative pathways [23, 24]. The reductions in IL-6, TNF-α, IL-1β, MCP-1, and RANTES are in line with persistent cytokine dysregulation reported in ART-treated PLWH and with the recognized contribution of peripheral immune activation to CNS injury [32–35, 38–41]. Likewise, the mitigation of glutamate elevations aligns with extensive evidence implicating excitotoxicity in HAND [47], and the reduction of 8-OHdG corroborates the role of oxidative DNA damage in HIV-associated neurodegeneration [7, 48–50]. Taken together, these converging lines of evidence independently support and strengthen the results of the present study.

Several limitations should be acknowledged. First, the study was conducted in a murine model, which, although representative of key neuropathological features of HAND, may not fully reflect the complexity of the disorder in PLWH. Second, the duration of treatment was relatively short; therefore, the long-term efficacy, safety, and potential toxicity of chronic EVG + CUR administration remain unaddressed. Third, while the study demonstrated synergistic therapeutic benefits, the precise molecular mechanisms underlying the EVG-CUR interaction—particularly those involving mitochondrial function, autophagy, and neurotrophic signaling—have not yet been elucidated. Fourth, we did not measure EcoHIV viral load, and thus cannot determine the extent to which EVG + CUR altered viral burden. Future studies will incorporate quantitative viral assays across brain regions and plasma and correlate these with behavioral and cytokine/oxidative endpoints to delineate antiviral versus neuroimmune contributions to efficacy. Fifth, our oxidative-stress assessment relied on a single biomarker (8-OHdG); we did not measure lipid peroxidation (e.g., MDA/4-HNE), protein oxidation (carbonyls), or antioxidant capacity (TAC; enzymatic defenses such as SOD, catalase, and GPx). Future studies will include this broader panel and correlate it with behavioral and cytokine outcomes to delineate the specific redox pathways engaged by EVG + CUR.

## Conclusion

This study provides the first preclinical evidence that combination therapy with EVG and CUR effectively mitigates EcoHIV-induced cognitive and motor deficits, neuroinflammation, and oxidative stress, while preserving CNS homeostasis. By concurrently targeting viral persistence, neuroinflammatory signaling, and oxidative injury, EVG + CUR offers a multifaceted therapeutic approach for HAND. The superior efficacy observed with IN administration underscores the importance of optimizing CNS-targeted drug delivery strategies. Together, these findings support the potential of EVG + CUR as a promising adjunctive treatment strategy for managing HAND beyond current ART approaches.

Future studies should focus on validating the long-term safety and therapeutic efficacy, including other behavioral domains like anxiety-like and depressive phenotypes, of intranasal EVG + CUR combination therapy in non-human primate models and clinical settings to enhance translational relevance. Future studies should also focus on mechanistic studies exploring the role of the combination therapy on mitochondrial dynamics, epigenetic regulation, and intracellular signaling pathways, which would likely provide novel druggable target(s) for HAND treatment.

### Statistical analysis

All data are expressed as mean ± standard error of the mean (SEM). Statistical analyses were performed using GraphPad Prism version 10 (GraphPad Software, San Diego, CA, United States). One-way or two-way analysis of variance (ANOVA) followed by Tukey’s multiple comparisons test was used to assess differences between groups, as appropriate. A p-value of ≤0.05 was considered statistically significant. Specific tests and significance levels are indicated in the corresponding figure legends.

## Data Availability

The original contributions presented in the study are included in the article/Supplementary Material, further inquiries can be directed to the corresponding author.
