# Supplementary material for: Curcumin-enhanced elvitegravir therapy mitigates neuroinflammation and cognitive deficits in EcoHIV mice
Source: Exp Biol Med (Maywood). 2025 Nov 7;250:10758. doi: 10.3389/ebm.2025.10758 (PMC12634458; doi:10.3389/ebm.2025.10758)

**Curcumin-Enhanced Elvitegravir Therapy Mitigates Neuroinflammation and Cognitive Deficits in EcoHIV Mice**

**AUTHOR NAMES.** Sandip Godse^1^, Lina Zhou^1^, Namita Sinha^1^, Mohd Salman^2^, Tauheed Ishrat^2^, Santosh Kumar*,^1^.

**AUTHOR ADDRESS.**

^1^Department of Pharmaceutical Sciences, University of Tennessee Health Science Center, 881 Madison Ave, Memphis, TN 38163, USA

^2^Department of Anatomy and Neurobiology, College of Medicine, The University of Tennessee Health Science Center, 875 Monroe Avenue, Memphis, TN, 38163, USA

*Corresponding author

**Table S1. CatWalk XT® Automated Gait Analysis Parameters and Their Descriptions**

| Parameter | Description |
| --- | --- |
| Run Duration | Total time taken to complete a run across the walkway. |
| Average Speed | Average walking speed during a run (cm/s). |
| Cadence | Number of steps taken per second. |
| Stride Length | Distance between two consecutive placements of the same paw (cm). |
| Swing Time | Duration when a paw is in the air between steps (s). |
| Stance Time | Duration a paw remains in contact with the walkway during a step (s). |
| Step Cycle | Total duration of one stride cycle (s). |
| Base of Support (BOS) | Lateral distance between forelimbs or hindlimbs (cm), indicating stability. |
| Print Area | Contact area of the paw with the glass surface during stance (cm^2^). |
| Print Intensity | Light intensity reflecting the force applied by the paw (arbitrary units). |
| Print Length | Longitudinal length of the paw print (cm). |
| Print Width | Width of the paw print (cm). |
| Maximum Contact | Timing of the maximal paw contact during stance phase (% of stance). |
| Swing Speed | Speed at which the paw swings forward (cm/s). |
| Run Maximum Variation | Variability in run speed, reflecting gait inconsistency. |
| Step Sequence Pattern | Order in which paws are placed (Cruciate, Alternate, Rotate patterns). |
| Regularity Index | Percentage of steps forming normal step sequence patterns (100% = perfect coordination). |
| Support Type Durations | Time spent with various combinations of paws in contact (single, diagonal, lateral, or all four paws). |
| Phase Dispersions | Timing difference between paw placements relative to a reference paw (% of stride cycle). |
| Paw Contact Timing | Timing of initial paw contact, maximum contact, and lift-off. |

**Supplementary Figures**


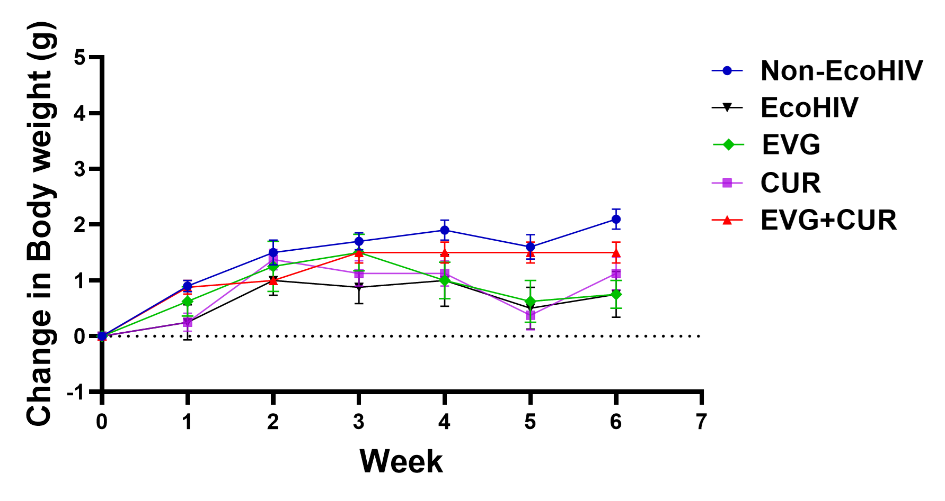


**Figure S1**: Body weight changes versus baseline weight throughout 6 weeks of the experiment. Two-way ANOVA test was applied to compare between multiple groups. Data are presented as mean ± SEM (n = 8).

**Figure S2: Figure 5 original blots E, F, G, H, and I.**

Western blot analysis of neural protein markers across different treatment groups in mice brain


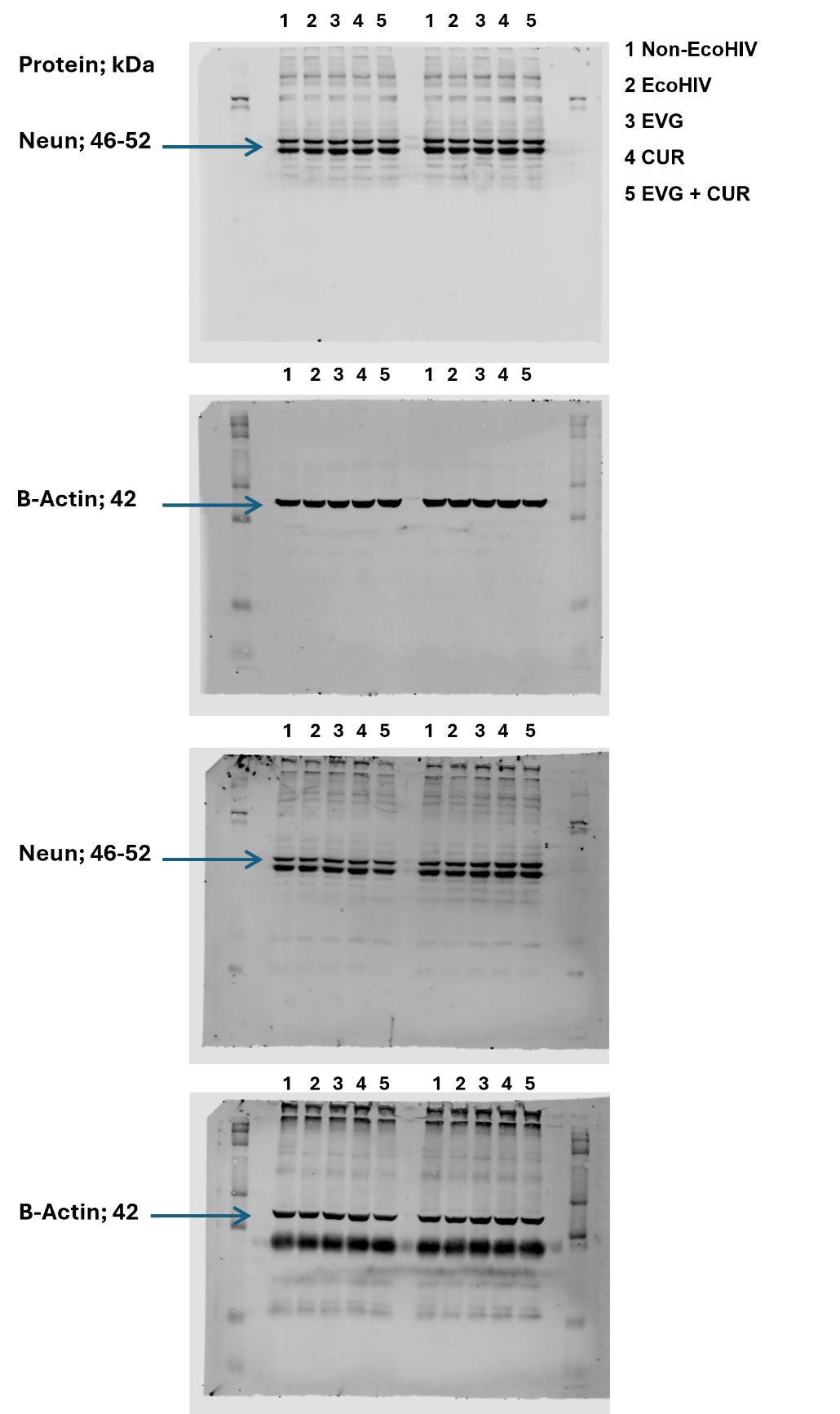


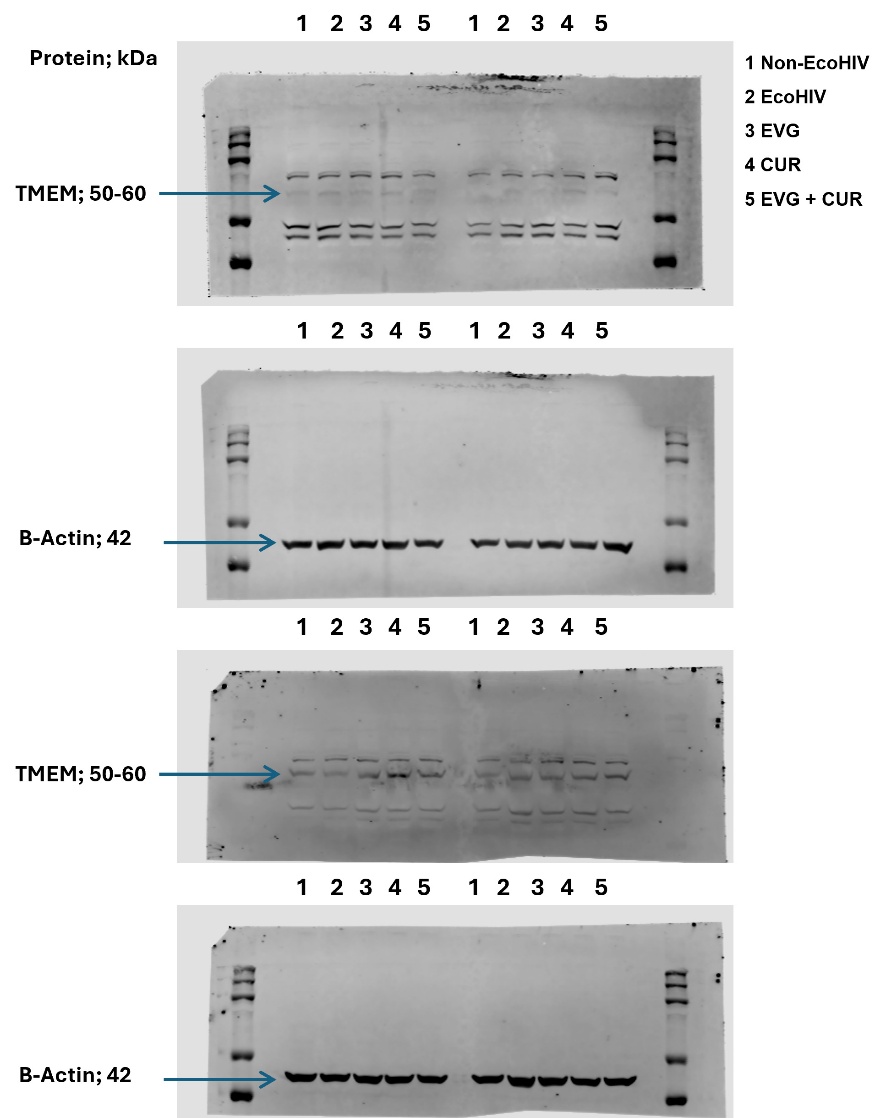


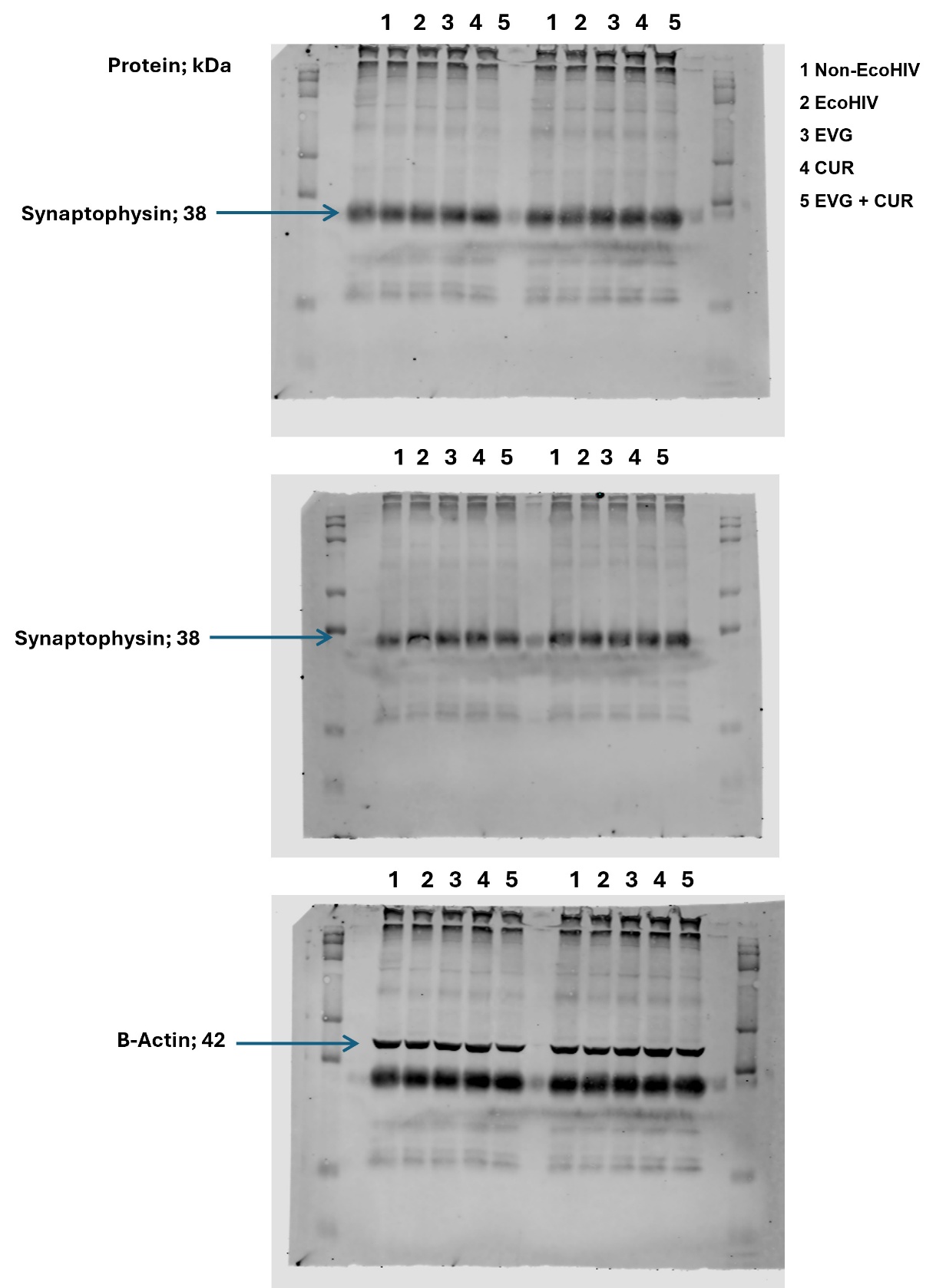

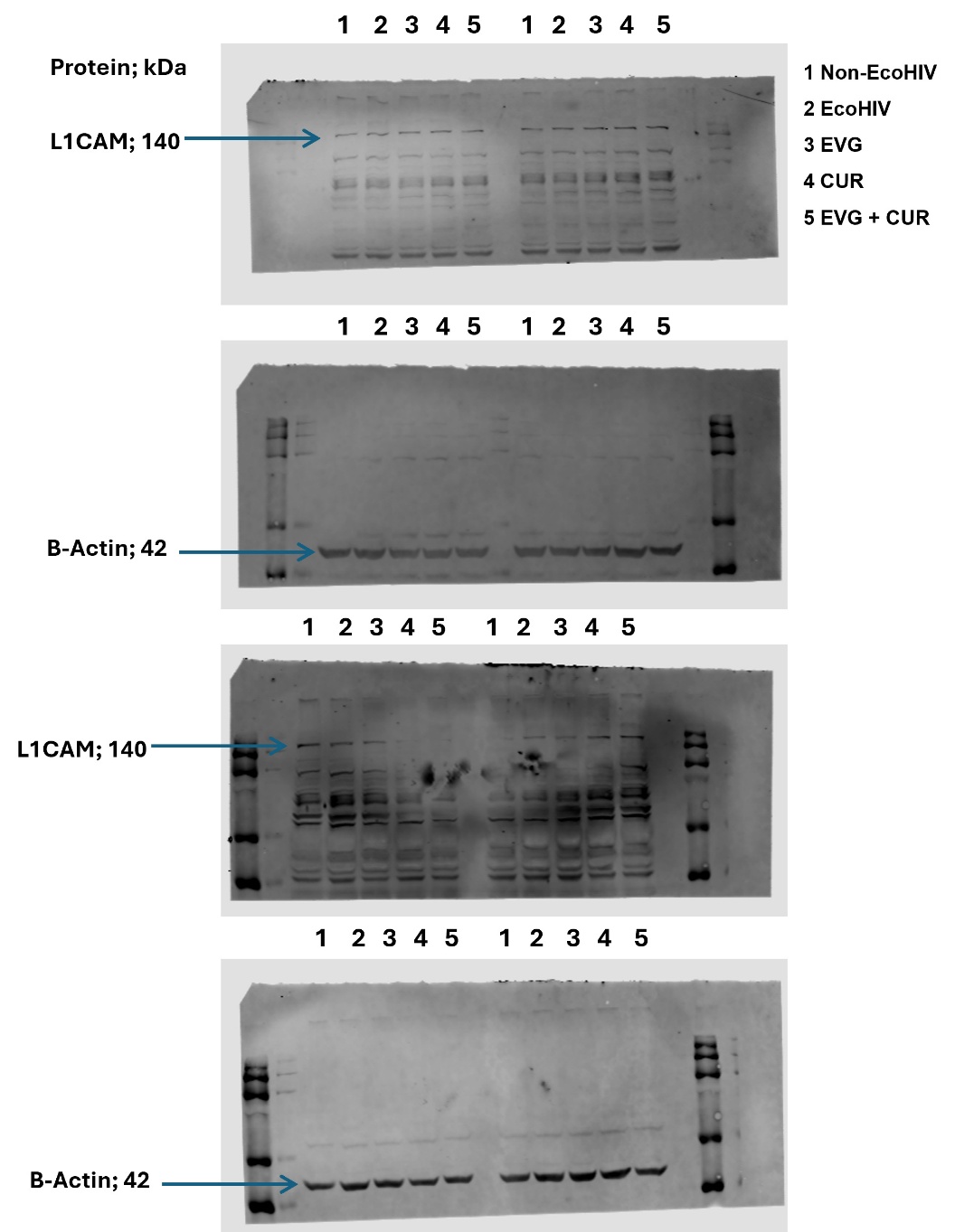

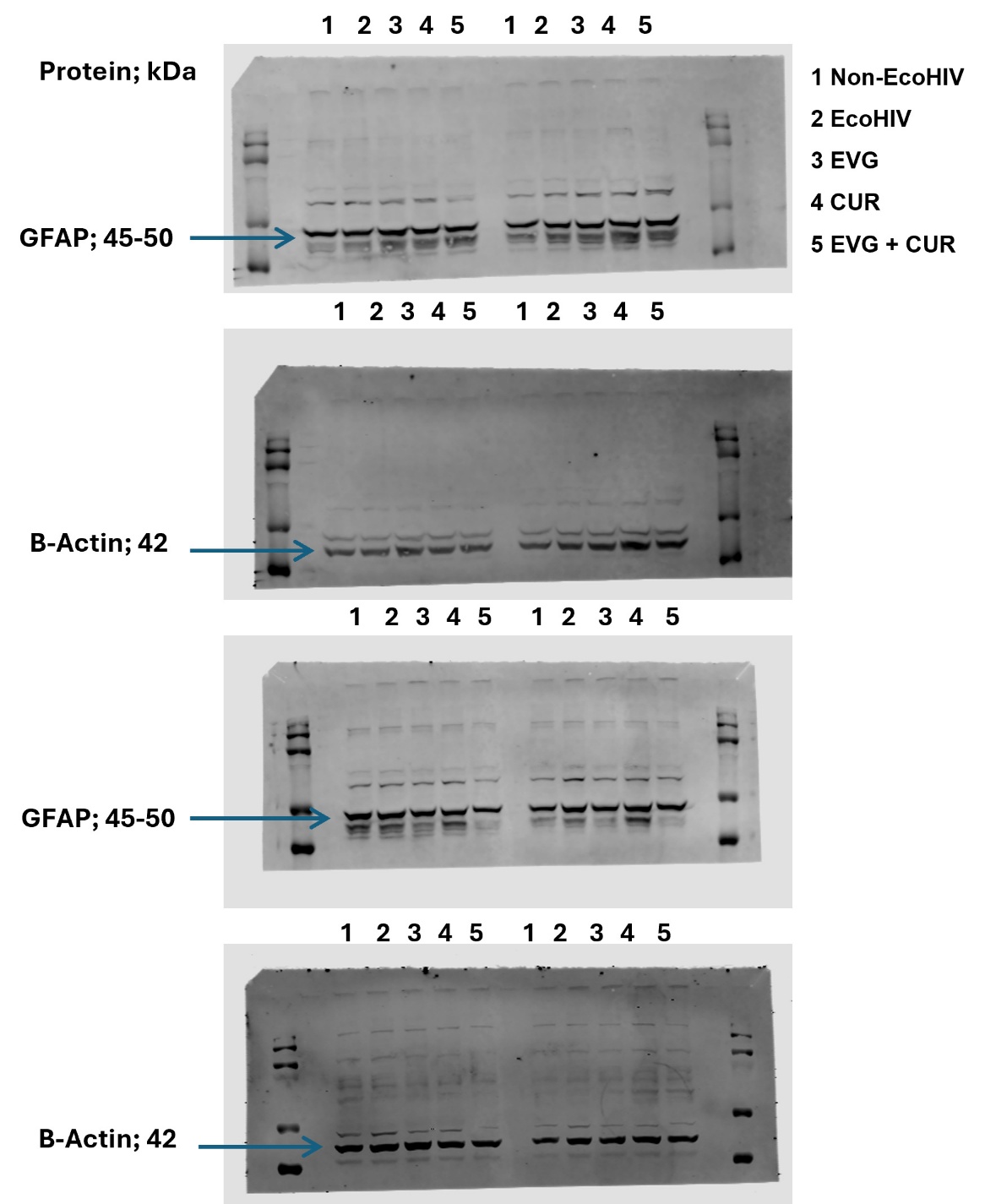

Supplement: Supplementary file 1 [file DataSheet1.docx]
